# Optimization of Goat Milk with ACE Inhibitory Peptides Fermented by *Lactobacillus bulgaricus* LB6 Using Response Surface Methodology

**DOI:** 10.3390/molecules22112001

**Published:** 2017-11-21

**Authors:** Guowei Shu, Xiaoyu Shi, He Chen, Zhe Ji, Jiangpeng Meng

**Affiliations:** 1 School of Food and Biological Engineering, Shaanxi University of Science and Technology, Xi’an 710021, China; shuguowei@gmail.com (G.S.); chenhe419@gmail.com (H.C.); somber-sky@163.com (Z.J.); 2 Department of Research and Development, Xi’an Baiyue Gaot Milk Corp., Ltd., Xi’an 710089, China; byjpmeng@gmail.com

**Keywords:** goat milk, fermentation conditions, ACE inhibitory activity, Box–Behnken design

## Abstract

In the present study, the incubation conditions of goat milk fermented by *Lactobacillus bulgaricus* LB6 were optimized to increase the angiotensin converting enzyme (ACE, EC 3.4.15.1) inhibitory activity by Box–Behnken design of response surface methodology. Incubation temperature, whey powder, and calcium lactate had significant effects on ACE inhibition rate and viable counts of LB6 during incubation. The results showed that optimal conditions of fermentation were found to be 37.05 °C, 0.8% (*w*/*w*) whey powder and 0.50% (*w*/*w*) calcium lactate. ACE inhibition rate increased significantly from 71.04 ± 0.37% to 83.31 ± 0.45% and the viable counts of *Lactobacillus bulgaricus* LB6 reached to 8.03 × 10^7^ cfu·mL^−1^ under the optimal conditions, which approached the predicted values 83.25% and 8.04 × 10^7^ cfu·mL^−1^. The optimal fermentation conditions can be a good reference for preparing ACE inhibitory peptides from goat milk.

## 1. Introduction 

Hypertension is a major risk factor for myocardial infarction, heart failure, stroke, aneurysm, arteriiosclerosis, renal disease, and peripheral arterial disease and it may lead to chronic kidney disease as well [1,2,3]. Six categories of drugs (diuretics, calcium channel blockers, angiotensin II receptor blockers, ACE inhibitors, α-adrenergic antagonist, and β-blockers) are employed for anti-hypertension [4]. ACE inhibitors are more widely applied due to the protection to target organ without adverse effects on the metabolism of glycolipid.

ACE, also known as Kinase II, is a metalloproteinase containing two zinc-catalytically active sites [5], which can affect both the renin–angiotensin system (RAS) and the kallikrein–kinin system (KKS) simultaneously. Inactive angiotensin-I is hydrolyzed to angiotensin-II with effective vasoconstrictor action in the RAS system under the combined action of renin and ACE, leading to elevated blood pressure. The KKS system is an endogenous blood pressure system in which the ACE inhibits the antihypertensive effect by inactivating the bradykinin [6,7,8,9,10]. The role that ACE plays in these two systems ultimately resulted in the elevation of blood pressure.

ACE inhibitors can inhibit ACE activity. Since the side effects such as dry-cough, pruritic rash, and taste disorders occurred when using synthetic ACE inhibitors [11,12], studies on food-derived ACE inhibitory peptides to produce antihypertensive products without toxic side effects have shown much necessity. ACE inhibitory peptides can be integrated with ACE tightly in the human body and it is not easy to release them from the ACE binding area. There are competitive inhibitory effects between ACE inhibitory peptides with both angiotensin-I and bradykinin in the process of blood pressure regulation [13], which can prevent the formation of angiotensin-II and inactivation of bradykinin at the same time. Thus, ACE inhibitory peptides can be used for anti-hypertension.

The food-derived ACE inhibitory peptides, which have been reported, are mainly derived from animal proteins, vegetable proteins, and algae. Milk protein—including casein, lactalbumin and lactoglobulin—is a kind of source of ACE inhibitory peptides for animal protein sources [14,15]. Goat milk has an unparalleled advantage compared to other dairy. The protein, fat, minerals, vitamins, and other nutrients of goat milk are more abundant than other milk [16,17,18]. Moreover, goat milk overall protein particles are smaller, composed of short-chain fatty acids. Goat milk is closer to breast milk than other dairy, and even has a higher level of immunoglobulin than breast milk [19]. Lactic acid bacteria are a group of gram-positive bacteria that translate carbohydrates into lactic acid, which have been widely used in food fermentation. Proteins are hydrolyzed into plenty of bioactive peptides with some health care functions by lactic acid bacteria during incubation. There are 70 kinds of ACE inhibitory peptides from goat milk in the AHTPDB [20]. Parmar et al. [21] identified and characterized 26 peptides with ACE inhibitory activity from fermented goat milk with *Lactobacillus casei* NK9 and *Lactobacillus fermentum* LF. Sixteen peptides in fermented milk beverage made from caprine milk were identified by Quiro’s et al. [22]. Two of these peptides, with sequences PYVRYL and LVYPFTGPIPN, showed potent ACE inhibitory properties.

In our previous work, four strains—including *L. bulgaricus, Lactobacillus rhamnosus, Lactobacillus helveticus* and *Lactobacillus reuteri*—were selected as starters of ACE inhibitory peptides among 28 probiotic *Lactobacillus* strains [23]. The effects of incubation conditions of *Lactobacillus bulgaricus* LB6 on the ACE inhibition rate and viable counts in fermented goat milk were performed by single factor experiments [24]. A Plackett–Burman designed experiment was conducted subsequently. Incubation temperature, whey powder, and calcium lactate were selected as main factors, which had positive effects on ACE inhibition rate with a low level (30 °C, 0.6%, 0.4%) and a high level (37 °C, 0.8%, 0.5%). According to the results of the steepest ascent experiment, 37 °C, 0.8% whey powder and 0.5% calcium lactate were set as the centers of Box–Behnken design (data unpublished). The aim of this study was to optimize the levels of the significant factors (incubation temperature, whey powder and calcium lactate) for incubation conditions of *L. bulgaricus* LB6 to maximize ACE inhibitory activity as well as viable counts during fermentation by using Box–Behnken design based on the previous study [25].

## 2. Results

### 2.1. The Experimental Design and Results of Box–Behnken

RSM (Response surface methodology) was carried out to determine the optimum concentrations of the three significant factors for fermented goat milk, incubation temperature (A), whey powder (B), and calcium lactate (C). The factors and levels are given in Table 1. The experimental design and results of Box–Behnken for incubation conditions of LB6 are shown in Table 2. The ACE inhibition rate is represented by R_1_ (%) and viable counts of LB6 is represented by R_2_ [lg(cfu·mL^−1^)].

### 2.2. Regression Analysis

The data were analyzed to get quadratic regression model by using Design Export based on the data from the Box–Behnken experiment. A multiple regression equation correlating the response function with the independent variables could be obtained as:

R_1_ = 83.26 + 0.25A + 0.18B − 0.022C + 0.26AB + 0.43AC + 0.19BC − 5.69A^2^ − 3.43B^2^ − 1.63C^2^

R_2_ = 8.04 + 0.0075A − 0.00875B − 0.024C − 0.0075AB + 0.038AC + 0.015BC − 0.20A^2^ − 0.16B^2^ − 0.043C^2^

where: R_1_ and R_2_ are corresponding expected value of ACE inhibition rate and viable counts of LB6. A, B and C are the coded values of the independent variables, incubation temperature, whey powder, and calcium lactate, respectively.

### 2.3. Variance Analysis

Analysis of variance (ANOVA) is used to determine whether the model is valid according to the significance. ANOVA for response surface of ACE inhibition rate and viable counts of LB6 were shown in Table 3.

As shown in Table 3, the probability value of ACE inhibition rate indicated a high significance for regression equation (*p* = 0.0007 < 0.001) and the lack of fit was insignificant (*p* = 0.3878 > 0.05). It revealed the effectiveness of regression analysis, which suggested that the regression model could be used to fit the effect of three factors on ACE inhibition rate. The difference between adjusted R-squared and predicted R-squared was 0.16, within the value of 0.2, which implied that the model was highly significant. Furthermore, the value of determination coefficient (R^2^ = 98.24%) was calculated. It suggested that a 98.24% response to the ACE inhibition rate was caused by changing in the concentration of A, B, and C and their interactions.

The ANOVA showed the model for viable counts of LB6 with a *p*-value was less than 0.05, which was significant in Table 3. The lack of fit (*p* = 0.0939 > 0.05) was insignificant. Therefore, the model equation was corroborated to be a suitable model to describe the value of the viable counts for LB6. The value of determination coefficient, which was 98.27%, indicated that only 1.73% of the variability in the viable counts of LB6 could not be explained by the predicted equation of model. AB and BC showed weak mutual interactions on the effect of viable counts for LB6, while the interaction of AC was significant (*p* = 0.0488 < 0.05).

**Figure 1:**
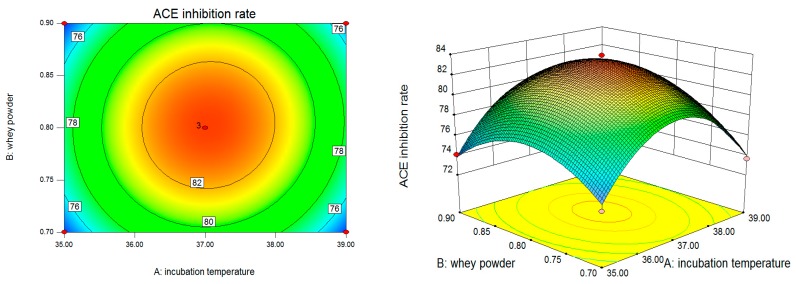

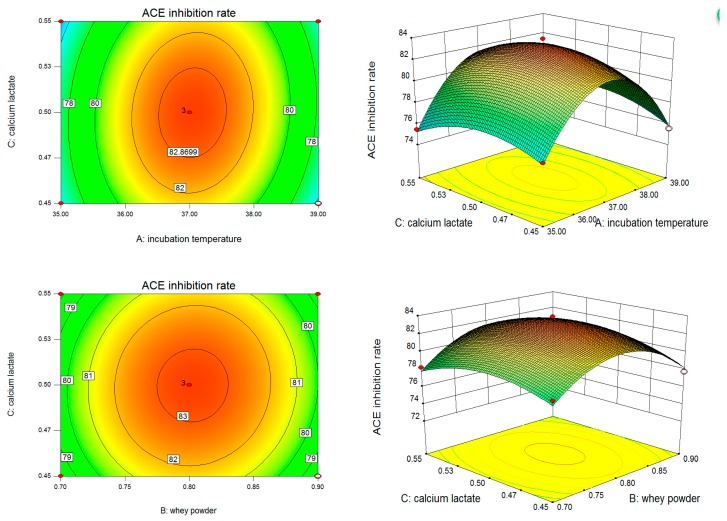
Contour plots and response surface plots of incubation temperature A, whey powder B, calcium lactate C to ACE inhibition rate (R_1_).

Two-dimensional contours revealed that ACE inhibition rate changed with the changes of temperature, whey powder, and calcium lactate and their corresponding three-dimensional response surface were generated to determine the interaction of the three variables on the corresponding variables better (Figure 1). As shown in Figure 1, the contour plots seemed to be elliptical or nearly circular. It implied that AB, AC, and BC had mutual interactions for the ACE inhibition rate, while they were weak (*p*_AB_ = 0.5161 > 0.05, *p*_AC_ = 0.2995 > 0.05, *p*_BC_ = 0.6435 > 0.05). Therefore, the effect of one factor on ACE inhibition rate was dependent on the level of another one. The quadratic main effects of incubation temperature, whey powder, and calcium lactate were significant (*p*_A_^2^ < 0.001, *p*_B_^2^ = 0.0003 < 0.001, *p*_C_^2^ = 0.0087 < 0.01), which suggested that there was not a simple linear correlation between the variables and ACE inhibition rate.

The maximum responses values of ACE inhibition rate 83.25% and the viable counts of *L. bulgaricus* LB6 8.03 × 10^7^ cfu·mL^−1^ were obtained at 37.05 °C, 0.8% whey powder, and 0.5% calcium lactate as predicted, respectively. The design of verification experiment was dependent on the optimization results (A = 37.0 °C, B = 0.8%, C = 0.5%). The results demonstrated that the ACE inhibition rate and viable counts of LB6 were 83.31 ± 0.45% (*n* = 3) and 8.03 × 10^7^ cfu·mL^−1^ (*n* = 3) under the optimum conditions. There was no significant difference from the predicted value, indicating suitability of the model. The control had values of ACE inhibition rate as 71.04 ± 0.37% and viable counts as 4.53 × 10^7^ cfu·mL^−1^ at 37 °C without addition of whey powder and calcium lactate. The ACE inhibition rate was improved by 12.27% using RSM optimization compared to the control. The viable counts of LB6 was increased significantly from 4.53 × 10^7^ cfu·mL^−1^ to 8.03 × 10^7^ cfu·mL^−1^.

## 3. Discussion

It had been reported that great quantities of optimum methods were used to release more ACE inhibitory peptides and the approaches to enhance ACE inhibitory activity in fermented products have recently received considerable attention. B vitamins were added to the soybean milk fermentation medium and the ACE inhibitory activity had significant improvements compared to control. The addition of B vitamins enhanced protein hydrolysis degree, which resulted in more bioactive peptides being released [26]. Prebiotics have the same promotion effects as B vitamins. A study by Yeo et al. [27] showed that the addition of prebiotics, such as inulin and pectin, enhanced the antihypertensive effect in probiotic-fermented soymilk.

Temperature was testified to be a significant factor to fermented milk, which could owe to the influence on the growth of *L. bulgaricus* and the activity of proteolytic enzyme. *L. bulgaricus* LB6 exhibited extremely poor growth with a weak metabolic activity when the temperature was low, causing low values of ACE inhibition rate and viable counts of LB6. With the increase of temperature, *L. bulgaricus* grew quickly and the enzyme activity increased gradually. Therefore, the activity of LB6 and the ACE inhibition were enhanced. The activity of proteolytic enzymes was destroyed with temperature elevation, causing the gradual decrease of ACE inhibition rate.

Whey powder has been used to improve production of ACE inhibitory peptides in a suitable concentration. The viable counts of LB6 and ACE inhibition rate in fermented goat milk increased first and then decreased as the concentration of whey powder increasing. However, the ACE inhibition rate gradually lessened in the cow milk fermented by *Lactobacillus casei* with increasing concentrations of whey powder [28], which may due to the structure and content of whey protein in bovine and goat milk are different.

Low concentrations promote ACE inhibitory activity, while high concentration suppression of ACE inhibitory activity is also applicable to calcium lactate. It was reported that calcium dihydrogen phosphate was added when preparing rapeseed ACE inhibitory peptides by liquid-state fermentation conditions and the ACE inhibition rate was greatly improved [29]. The effects of ionic calcium on *L. casei* and *Bifidobacterium bifidum* were studied by Gonzalez et al. [30]. It was found that ACE inhibitory peptides had a higher yield with the calcium added, which might be related to the effect of ionic calcium channel by speculation.

The ACE inhibition rate and viable counts of LB6 can be significantly increased by optimizing incubation conditions of fermented goat milk using RSM. The ACE inhibition rate improved from 71.04 ± 0.37% to 83.31 ± 0.45% in present study, which provides a reference for improving the production of ACE inhibitory peptides by *L. bulgaricus* fermentation.

The effect of fermentation conditions on the ACE inhibitory activity in milk fermented by yeast, *Kluyveromyces marxianus*, was investigated by Li et al. [31] using RSM. Optimal conditions of incubation were found to be temperature 32 °C, initial pH 6.5, 6% (*v*/*v*) inoculum level and rotation speed of 189 rpm. ACE inhibition rate reached 81.23 ± 2.69% under optimal conditions. It is lower than the result of this study, which may be due to that *Lactobacillus* proteolytic enzyme is superior to yeast. Pan et al. [32] found that the maximum of ACE inhibition rate was 75.46 ± 0.42% when using *Lactobacillus helveticus* LB10 as starter culture to ferment milk under optimal conditions, which were 4% (*v*/*w*) inoculum, initial pH 7.5 of medium at 39.0 °C. Strains with high ACE inhibitory activity were screened by Chen et al. [23] and the result indicated that ACE inhibitory activity of *L. helveticus* was lower than that of *L. bulgaricus* under the same fermentation conditions, which was consistent with the results of this paper.

## 4. Materials and Methods

### 4.1. Strain

*L. bulgaricus* LB6 was provided by the college of Food and Biological Engineering, Shaanxi University of Science & Technology (Xi’an, China). The starter cultures were stored at −20 °C in freeze-dried powder. *L. bulgaricus* LB6 was activated successively three times in rehydrated Man Rogosa Sharpe broth (MRS, Hopebio, Qingdao, China) with 5% inoculum at 37 °C for 24 h prior use as starter culture to ferment goat milk.

### 4.2. Preparation of Fermented Goat Milk

Starter culture containing *L. bulgaricus* LB6 was inoculated into reconstituted skim goat milk pasteurized with 5% inoculum and fermented at 37 °C for 12 h.

### 4.3. Determination of ACE Inhibitory Activity

The whey fraction from the fermented goat milk was used to measure the ACE inhibitory activity. Aliquots of the fermented goat milk were collected, vigorously stirred, and centrifuged at 5000× *g* for 15 min to obtain the corresponding whey fractions. The supernatants collected were filtered through a Xinhua filter and used to determine their ACE inhibitory activity. ACE inhibitory activity was measured by a spectrophotometric assay according to the method of Cushman and Cheung (1971) with modifications. Added 80 μL of each sample to 200 μL sodium borate buffer (0.1 mol·L^−1^, pH 8.3) containing NaCl (0.30 mol·L^−1^) and hippuryl-histidyl-leucine (HHL) (5 mmol·L^−1^). ACE (20 μL, 0.1 U·mL^−1^) was added and incubate the reaction mixture at 37 °C for 30 min. 250 μL 1 mol·L^−1^ HCl was added to terminate the reaction. Hippuric acid formed was extracted by 1.7 mL ethyl acetate and evaporated at 120 °C for 30 min. The sample was dissolved into 2 mL deionized water after cooling at room temperature. The absorbance at 228 nm was measured in triplicate using UV-vis spectrophotometer (Shanghai Spectrum Instruments Co., Ltd., Shanghai, China).

### 4.4. Calculation of the ACE Inhibition Rate

ACE inhibition rate were determined by the following equation:

$$\mathrm{ACE}\;\mathrm{inhibition}\;\mathrm{rate}\;(\%)=\frac{X_{1}-X_{2}}{X_{1}-X_{3}}\times 100\%$$

where X_1_ is absorbance without the whey fraction, X_2_ is absorbance without ACE, and X_3_ is the absorbance in the presence of both ACE and the whey fraction.

### 4.5. Measurement of Viable Counts

Fermented goat milk was diluted serially by saline water (0.9%, *w*/*v*, NaCl) containing 0.1 g·L^−1^ peptone (0.9%, *w*/*v*, NaCl) and spread on MRS agar plates. The dilution in triplicate was incubated for 48 h at 37 °C. Results were expressed as colony forming units per milliliter (cfu·mL^−1^) of fermented milk by manual counting.

### 4.6. Optimization of Fermentation Conditions by RSM

RSM was employed for further optimization studies by determining the maximum response value and evaluation of the main effects, interaction effects, and quadratic effects. RSM of 3-variable and 3-level were used according to the previous study.

### 4.7. Validation of the Model

The parameters of incubation conditions were applied in further fermentation experiments based on the results of RSM. The experimental values were compared with predictive value to verify the effectiveness of the model.

### 4.8. Statistical Analysis of the Data

Design Expert software (Version 8.0.5, Stat-Ease. Inc., Minneapolis, MN, USA) was used for the analysis of the experimental data obtained and quadratic regression equation was established to analyze the response surface contour and surface plots.

## 5. Conclusions

Hypertension is a serious threat to human health and food-derived ACE inhibitory peptides can regulate high blood pressure without side effects. In this study, incubation conditions of fermented goat milk with *L. bulgaricus* LB6 were optimized by RSM to increase the production of ACE inhibitory peptides. The results showed that the maximum ACE inhibition rate was 83.31 ± 0.45% in the 0.8% (*w*/*w*) whey powder and 0.50% (*w*/*w*) calcium lactate at 37.05 °C. The viable counts of *L. bulgaricus* LB6 was reaching to 8.03 × 10^7^ cfu·mL^−1^ under the optimal conditions. Both ACE inhibition rate and viable counts were close to the predicted values, which indicated effectiveness of the model.

## Figures and Tables

**Table 1:** The factors level coding table of Box–Behnken for incubation conditions of LB6.

Factors	Coded Levels		
	−1	0	1
Temperature (°C)	35	37	39
Whey powder (%)	0.7	0.8	0.9
Calcium lactate (%)	0.45	0.50	0.55

**Table 2:** The experimental design and results of Box–Behnken for incubation conditions of LB6.

Runs	A	B	C	R_1_ (%)			R_2_ [lg(cfu·mL^−1^)]		
				Actual Value	Predicted Value	Residual	Actual Value	Predicted Value	Residual
1	−1	1	0	74.08	73.81	0.27	7.7	7.67	0.031
2	0	1	−1	77.77	78.21	−0.44	7.82	7.83	−0.015
3	−1	0	1	75.45	75.23	0.22	7.71	7.72	−0.014
4	0	1	1	78.04	78.53	−0.49	7.8	7.82	−0.018
5	0	0	0	83.19	83.26	−0.07	8.05	8.04	0.013
6	0	−1	−1	78.72	78.23	0.49	7.9	7.88	0.017
7	0	−1	1	78.25	77.81	0.44	7.82	7.81	0.015
8	1	0	−1	75.56	75.78	−0.22	7.8	7.79	0.014
9	1	1	0	75.49	74.83	0.66	7.67	7.67	0.00125
10	0	0	0	82.67	83.26	−0.59	8.03	8.04	0.00667
11	1	−1	0	73.68	73.96	−0.28	7.67	7.70	−0.031
12	−1	0	−1	76.31	76.15	0.16	7.83	7.85	−0.016
13	0	0	0	83.92	83.26	0.66	8.03	8.04	0.00667
14	−1	−1	0	73.32	73.98	−0.66	7.67	7.67	0.00125
15	1	0	1	76.44	76.60	−0.16	7.83	7.81	0.016

**Table 3:** ANOVA of response variables for ACE inhibition rate and viable counts of LB6.

Source	ACE Inhibition Rate				Viable Counts of LB6		
	DF	MS	F	Pr > F	MS	F	Pr > F
Model	9	17.58	31.10	0.0007 ***	0.0260	31.58	0.0007 ***
A	1	0.51	0.89	0.3880	0.0005	0.54	0.4967
B	1	0.25	0.44	0.5366	0.0006	0.73	0.4317
C	1	0.004	0.007	0.9358	0.0045	5.38	0.0680
AB	1	0.28	0.49	0.5161	0.0002	0.27	0.6265
AC	1	0.76	1.34	0.2995	0.0056	6.71	0.0488 *
BC	1	0.14	0.24	0.6435	0.0009	1.07	0.3476
A^2^	1	119.39	211.20	<0.0001 ***	0.1500	177.64	<0.0001 ***
B^2^	1	43.47	76.90	0.0003 ***	0.0930	110.41	0.0001 ***
C^2^	1	9.86	17.43	0.0087 **	0.0069	8.27	0.0348 *
Residual	5	0.57			0.0008		
Lack of fit	3	0.68	1.72	0.3878	0.0013	9.81	0.0939
Pure error	2	0.39			0.0001		
Cor Total	14						

*** *p* < 0.001, extremely significant; ** *p* < 0.01, very significant; * *p* < 0.05, significant. DF refers to degrees of freedom, MS refers to mean square, F and Pr > F refer to F and *p*-values, respectively.
